# A Cluster of Metabolic-Related Genes Serve as Potential Prognostic Biomarkers for Renal Cell Carcinoma

**DOI:** 10.3389/fgene.2022.902064

**Published:** 2022-07-07

**Authors:** Shuo Huang, Qihan Luo, Junhao Huang, Jiale Wei, Sichen Wang, Chunlan Hong, Ping Qiu, Changyu Li

**Affiliations:** School of Pharmaceutical Sciences, Zhejiang Chinese Medical University, Hangzhou, China

**Keywords:** prognostic biomarker, clear cell renal cell carcinoma, papillary renal cell carcinoma, metabolic-related genes, renal cance

## Abstract

Renal cell carcinoma (RCC) is the most common type of renal cancer, characterized by the dysregulation of metabolic pathways. RCC is the second highest cause of death among patients with urologic cancers and those with cancer cell metastases have a 5-years survival rate of only 10–15%. Thus, reliable prognostic biomarkers are essential tools to predict RCC patient outcomes. This study identified differentially expressed genes (DEGs) in the gene expression omnibus (GEO) database that are associated with pre-and post-metastases in clear cell renal cell carcinoma (ccRCC) patients and intersected these with metabolism-related genes in the Kyoto encyclopedia of genes and genomes (KEGG) database to identify metabolism-related DEGs (DEMGs). GOplot and ggplot packages for gene ontology (GO) and KEGG pathway enrichment analysis of DEMGs with log (foldchange) (logFC) were used to identify metabolic pathways associated with DEMG. Upregulated risk genes and downregulated protective genes among the DEMGs and seven independent metabolic genes, RRM2, MTHFD2, AGXT2, ALDH6A1, GLDC, HOGA1, and ETNK2, were found using univariate and multivariate Cox regression analysis, intersection, and Lasso-Cox regression analysis to establish a metabolic risk score signature (MRSS). Kaplan-Meier survival curve of Overall Survival (OS) showed that the low-risk group had a significantly better prognosis than the high-risk group in both the training cohort (*p* < 0.001; HR = 2.73, 95% CI = 1.97–3.79) and the validation cohort (*p* = 0.001; HR = 2.84, 95% CI = 1.50–5.38). The nomogram combined with multiple clinical information and MRSS was more effective at predicting patient outcomes than a single independent prognostic factor. The impact of metabolism on ccRCC was also assessed, and seven metabolism-related genes were established and validated as biomarkers to predict patient outcomes effectively.

## 1 Introduction

As the third most lethal tumor of the urinary system after prostate cancer and bladder cancer, renal cell carcinoma (RCC) is getting more attention (Global Burden of Disease Cancer et al., 2022). There are almost 430,000 new RCC patients worldwide each year, of whom approximately 180,000 will die of this disease (Sung et al., 2021). The prevalence and mortality of RCC continue to rise, posing a severe threat to human health (Owens, 2016; Turajlic et al., 2018; Elias et al., 2021).

RCC is a general term for a class of diseases divided into different pathological subtypes based on histological and morphological differences. The most common RCC subtype, clear cell renal cell carcinoma (ccRCC), accounts for approximately 75% of cases, followed by papillary renal cell carcinoma (pRCC), accounting for 18.5% of cases. Other rare subtypes of RCC include renal chromophobe carcinoma and renal collecting duct carcinoma (Ricketts et al., 2018). Recent studies have demonstrated metabolic abnormalities in a large proportion of RCC cases (Linehan et al., 2019; Bobulescu et al., 2021; Qi et al., 2021).

As imaging diagnostic technology and treatment methods continue to develop, it becomes easier to detect tumors early and initiate timely treatment, improving the survival rate of RCC patients. As a result, the proportion of patients with advanced RCC has declined from 30 to 17% (Strizova et al., 2019; Schulz et al., 2021; Tariq et al., 2022). While many small-molecule targeted drugs with immune checkpoint inhibitors have been marketed and put into use, side effects and drug resistance limit their application. In addition, a large proportion of patients receiving immune checkpoint inhibitor therapy have a similar prognosis as those receiving targeted agents. Notably, the prognosis of metastatic RCC patients is still poor, with the 5-years survival rate remaining at 10–15%. Thus, there is an urgent need to find reliable biomarkers to predict RCC patient outcomes (Kabaria et al., 2016; Reed et al., 2019; Lee et al., 2021a; Bedke et al., 2021; Doppalapudi et al., 2021; Ince and Eisen, 2021).

Metabolism is the most fundamental biological process of organism self-renewal. Tumor cells have different metabolic processes than normal cells because they derive most of their energy from glycolysis while normal cells obtain energy through oxidative phosphorylation under normoxic conditions (Fang et al., 2021; Huang et al., 2021; Morrissey et al., 2021; Xing et al., 2021). Using ccRCC as an example, studies have shown that loss of VHL gene function or VHL gene loss on the 3P chromosome exists in more than 90% of hereditary and a large proportion of sporadic ccRCC (Jonasch et al., 2021). This weakens the degradation of targeted HIF1/2 transcription factors, resulting in the accumulation of HIF1/2 transcription factors under normoxic conditions, putting cells in pseudo-hypoxic situations that lead to the remodeling of metabolic processes and upregulation of various growth factors (Bacigalupa and Rathmell, 2020). Metabolic remodeling of the tumor causes metabolic abnormalities at the original location (Li et al., 2014). In addition, tumor cells use exosomes and cytokines to alter cell metabolism in other body regions, weakening the immune response and enabling tumor metastasis (Morrissey et al., 2021).

Since abnormal cell metabolism is an essential marker for tumorigenesis and progression, it was hypothesized that metabolism-related genes could predict RCC patient outcomes (Wettersten et al., 2017). Many studies have assessed the relationship between metabolic markers and outcomes associated with the ccRCC subtype (Hakimi et al., 2016; Wu et al., 2020; Zhang et al., 2021). In contrast, few have explored the relationship between metabolic prognostic markers and other significant RCC subtypes. This study investigated the correlation between the metabolic machinery and the prognosis of ccRCC patients using the Gene Expression Omnibus (GEO, https://www.ncbi.nlm.nih.gov/gds/?term) (Barrett et al., 2013) and the Cancer Genome Atlas (TCGA, https://www.cancer.gov/tcga) databases. A metabolic gene-based risk score signature was established to predict ccRCC patient outcomes effectively. The signature was further validated using pRCC patient data to reveal potential associations between the major subtypes of RCC.

## 2 Materials and Methods

### 2.1 KEGG Metabolism-Related Genes Download

The KEGG database was searched using *Homo sapiens* as the species to identify genes that play a role in metabolic regulation. The number of metabolic pathways was obtained, and metabolic-related pathway-related genes were downloaded using the R package, KEGGREST (Therneau and Grambsch, 2000) and tidyverse (Wickham et al., 2019). All genes in the pathway were collected for further research (Supplementary Table S1).

### 2.2 GEO Genes Obtained

Gene expression data was downloaded from the GSE66272 dataset and divided into two subsets, GSE66270 (M0) and GSE66271 (M1), based on whether the tumor had metastasized. DEG screening was performed using the limma package (Ritchie et al., 2015) with the screening conditions set as: | log2FC | > 2 and adj. *p*-value <0.05. Visualization was performed using the ggplot2 package (Wickham, 2016) for the volcano plot. DEGs in the dataset were collected for further study.

### 2.3 Intersecting Genes by Venn Diagram

The DEGs were divided into the M0 and M1 groups based on the subset in which the genes were located and further subdivided into those with upregulated or downregulated expression. Upregulated DEGs in the M0 and M1 datasets were intersected, and the same action was taken for downregulated DEGs to obtain differentially expressed genes expressed before and after tumor metastasis. The upregulated and downregulated DEGs were each intersected with metabolism-related genes to obtain metabolism-related DEGs (MRGs) in tumors. Visualization was performed using Venny 2.1 (https://bioinfogp.cnb.csic.es/tools/venny/index.html).

### 2.4 Gene Ontology and Kyoto Encyclopedia of Genes and Genomes Pathway Enrichment Analysis and Protein-Protein Interaction Network Construction

MRGs’ GO and KEGG enrichment analysis was performed using the R package clusterprofiler (Wu et al., 2021), org. Hs.eg.db (Carlson, 2019) and GOplot (Walter et al., 2015) to assess biological processes and pathways in which genes play a role. Enrichment results were filtered by setting adj. *p*-value < 0.05 as condition, visualized by ggplot2. Protein functions can impact the function and expression of other proteins. Thus, protein-protein interaction (PPI) data were acquired using the STRING database. The filtering condition was set to the minimum required interaction score: highest confidence (0.900). Cytoscape3.8.0 was used to visualize the PPIs.

### 2.5 Identification of Metabolically Relevant Prognostic Genes in the TCGA-KIRC Cohort

RNAseq data in the level 3 HTSeq-FPKM format was downloaded from the ccRCC project (KIRC) in TCGA. The RNAseq data were converted into the resulting TPM format data, and Log2 (Exp+1) transformation and standardization processing were performed. After excluding abnormal data, univariate Cox regression analysis was performed using the survival package (Therneau, 2022).

The hazard ratio (HR) for selected genes from the univariate Cox regression analysis was visualized using the ggplot2 package with a *p*-value <0.05. The upregulated risk genes and downregulated protective genes were intersected with DEG Corresponding trends in change using the Venny2.1 tool for further analysis. A total of 23 differentially expressed metabolism genes (DEMGs) were selected. While an HR > 1 indicated that the gene may be a risk gene and be associated with a poor prognosis, an HR < 1 indicated that the gene may have a protective effect and be associated with a good prognosis.

Overall survival (OS) was defined as the time from the beginning of a random assignment to death for any reason (the last follow-up time was for patients lost to follow-up and the end of follow-up was for patients still alive when the study ended). OS is considered the most efficient endpoint in oncology clinical trials and is the preferred endpoint when patient survival can be adequately assessed. Disease-free survival (DFS) was defined as the time between the start of randomization and disease recurrence or death (for any reason). Progression-free interval (PFI) was defined as the time from the randomization date of primary treatment to disease recurrence. To increase the prediction accuracy, KM survival curves were plotted by Cox regression for DEMGs. The OS, DSS, and PFI of the DEMGs were analyzed, and the genes with a Cox regression *p*-value <0.05 were considered metabolism-related prognostic genes.

The Delong test and plotted receiver operating characteristic (ROC) curves were performed to validate the accuracy of the KM curves selected to predict the OS of ccRCC patients and determine whether the selected genes had potential as ccRCC biomarkers.

### 2.6 Establishment of a Metabolic Risk Score Signature (MRSS) for Prognosis

To select potential genes that are reliably associated with ccRCC prognosis, the glmnet package and survival package was used to fit the Least Absolute Shrinkage and Selection Operator (LASSO) regression model on 16 DEMGs. The study subjected parameter selection to a 10-fold cross-validation, with partial likelihood biases meeting the minimum criteria.

Subsequently, a multivariate Cox regression analysis was performed to obtain the regression coefficients of independent prognostic factors. The DEMGs with significant OS, DFS, and PFI survival curve differences were identified by combining multivariate Cox regression coefficients (β-Values), the MRSS model was built, and the formula was defined as follows (Exp represents the gene expression level and *β* represents regression coefficients from the multivariate Cox analysis):

$$\mathrm{MRSS}=\overset{i}{\underset{\;}{\sum }}\mathrm{ExpGen}e_{i}*\beta_{i}$$

Data processing was performed using the pROC package (Robin et al., 2011) and the area under the ROC curve (AUC) was calculated to test the accuracy of models for predicting 1, 3 and 5-years survival. Data were visualized using the ggplot2 package.

### 2.7 Metabolism Risk Score Signature Combined With Clinical Information

The TCGA dataset supplied clinical information, including the predictive prognosis factors. Univariate Cox analysis was performed to clarify the correlation between MRSS and OS. Multivariate Cox regression analysis was then used to evaluate whether the established MRSS could be an independent predictor. To evaluate the OS of ccRCC patients as comprehensively as possible, a prognostic nomogram including age, gender, stage and MRSS was created using the RMS package (Harrell, 2020) combined with the survival package. The concordance index (c-index) was used to evaluate the predictive accuracy of the nomogram.

### 2.8 Validation of the MRSS

To evaluate whether the model applies to other renal tumors, considering that they often have similar anatomic and pathological manifestations to ccRCC, pRCC (TCGA - KIRP) was selected as a validation dataset (*n* = 326).

Genes involved in building the MRSS mode were filtered out of the dataset and. Their expression was normalized; then, these data were combined with the MRSS calculation formula to calculate a risk score for each patient in the TCGA-KIRP cohort. Based on the median risk score, TCGA-KIRP patients were divided into a high-risk and a low-risk group. KM survival and ROC curves were plotted to assess differences in prognosis for the two groups of patients and whether there was sufficient accuracy in predicting outcomes using the MRSS.

The 1 -, 3 - and 5-years survival probabilities of TCGA-KIRP patients were also compared using a nomogram by combining age, gender, and disease stage. The calibration curve was plotted to verify the model’s performance, and the C-index was used to compare the accuracy of traditional TNM-stage, MRSS, and nomogram prediction.

To assess the prognostic value of the MRSS in ccRCC, the KM-plotter online analysis website (Lánczky and Győrffy, 2021) and the GEPIA database (Tang et al., 2017) contain multiple GEO/TCGA/GTEx datasets were used to plot survival curves. RNA expression of prognostic renal cancer-related genes was collected from the GENT2 website (Park et al., 2019) and visualized. The protein expression of the MRSS-involved genes was analyzed using the PDC000127 dataset from The National Cancer Institute’s Clinical Proteomic Tumor Analysis Consortium (Edwards et al., 2015) (CPTAC, https://proteomics.cancer.gov/programs/cptac).

### 2.9 Cell Culture

The 786-O ccRCC cell line was gifted by Prof Dahong Zhang from Zhejiang Provincial People’s Hospital. The HK-2 human normal renal tubular epithelial cell line was obtained from the cell bank of the Chinese Academy of Sciences. The 2 cell lines were cultured in Dulbecco’s Modified Eagle Medium (Gibco, United States) supplemented with 10% fetal bovine serum (Gibco, United States) and 1% penicillin-streptomycin (Gibco, United States) at 37 °C in an incubator (Thermo, United States) with 5% CO_2_ and saturated humidity.

### 2.10 RNA Extraction and Quantitative Real-Time Polymerase Chain Reaction

Total RNA of the 786-O and HK-2 cells was extracted using the Trizol reagent (Ambion, United States) and dissolved in RNase-free ddH2O (Takara Bio, China) under the manufacturer’s instructions. Next, the RNA samples were utilized for generating cDNA using the PrimeScript™ RT Master Mix Kit (Takara Bio, China). Finally, the cDNA samples were employed for qRT-PCR with the TB Green^®^ Premix Ex Taq™ Kit (Takara Bio, China). The amplification was performed using the CFX96 Real-Time system (BIO-RAD, United States). The primers of RRM2, MTHFD2, AGXT2, ALDH6A1, GLDC, HOGA1, ETNK2 and GAPDH were synthesized by Sangon (Sangon Biotech, China), and the sequences are listed in Supplementary Table S10. GAPDH was applied as an internal control, and the relative expression level of 7 genes was calculated by the 2^−ΔΔCT^ method (Schmittgen and Livak, 2008). The detailed procedure for qRT-PCR is given in Supplementary Table S11.

### 2.11 Statistical Analysis

Data were summarized and transformed using Excel. Independent prognostic factors were processed using univariate/multivariate Cox regression analysis. All data were further processed and visualized using R (v3.6.3), with a *p*-value <0.05 considered significant.

## 3 Results

### 3.1 Differentially Expressed Gene Analysis

**FIGURE 1:**
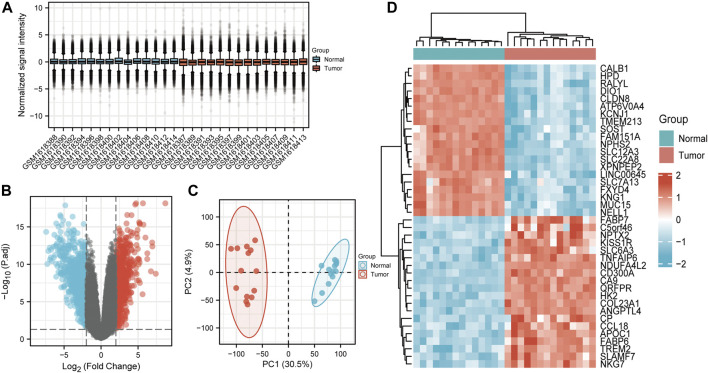
GSE66270 (M0) dataset analysis **(A)** Boxplots show that the median across samples is essentially on a horizontal line, indicating good normalization. **(B)** Volcano map; Blue and red dots represent up and downregulated genes that were eligible for screening. The current threshold was |logFC| > 2 with adj. *p*-value <0.05 **(C)** The samples from each group were separated in the PCA plot The ratio of PC1 and PC2 was high, indicating an obvious difference between groups and meaningful results of subsequent difference analyses should be reliable. **(D)** Heatmap of significantly differentially expressed genes.

**FIGURE 2:**
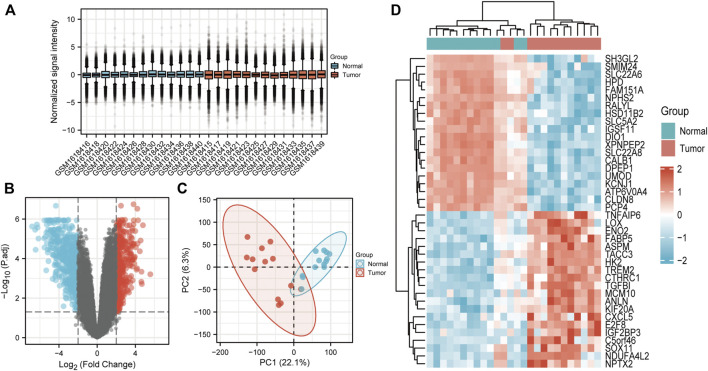
GSE66271 (M1) dataset analysis **(A)** Boxplots. **(B)** Volcano map; Blue and red dots represent up and downregulated genes. The current threshold was |logFC| >2 with a adj. *p*-value <0.05 **(C)** PCA plot indicated an obvious difference between the groups. **(D)** Heatmap of significantly differentially expressed genes.

**FIGURE 3:**
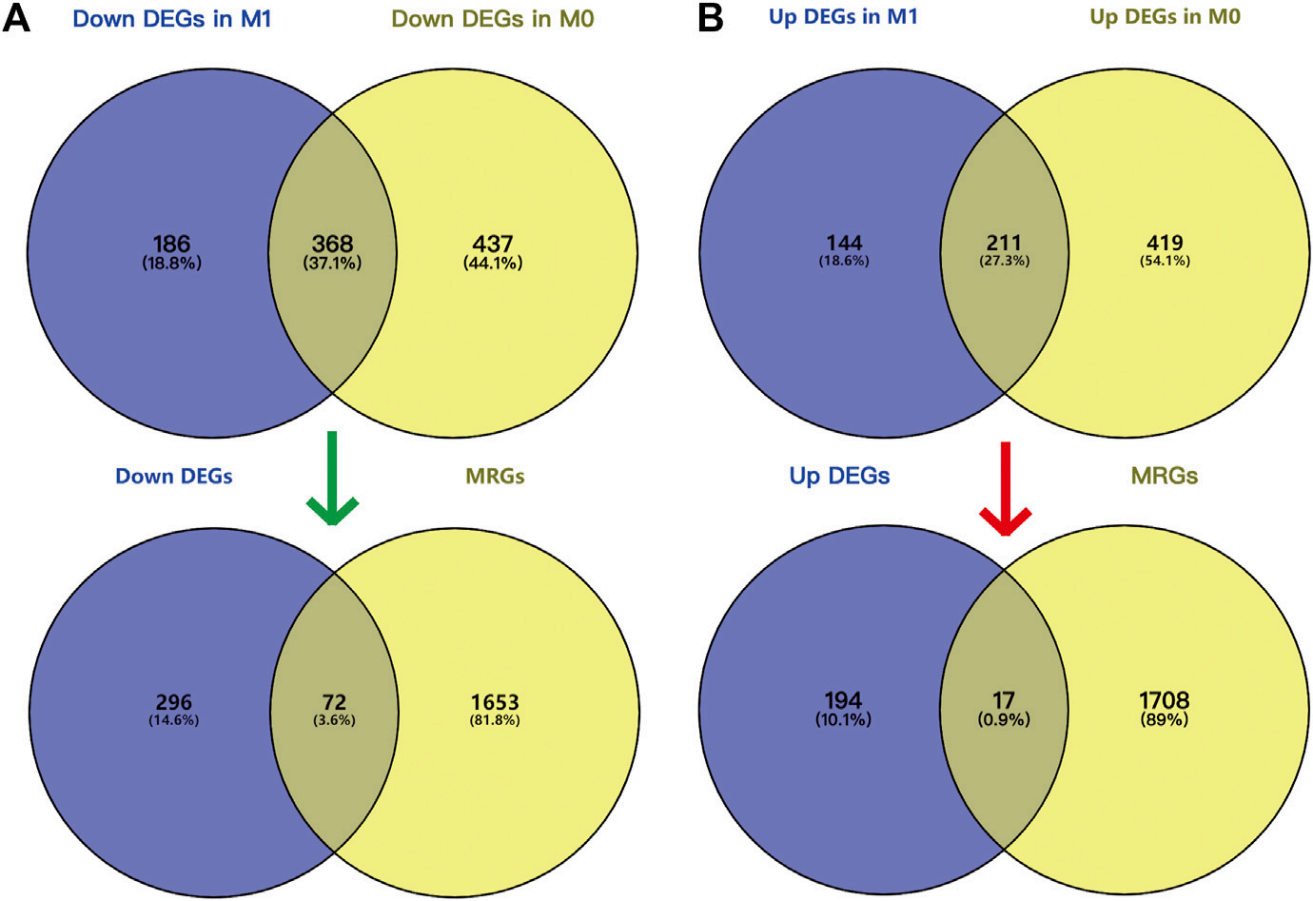
Screening DEMGs by intersecting DEGs and MRGs. Venn diagram of DEGs in M0 **(A)** and M1 **(B)** datasets and MRGs. GO and KEGG pathway enrichment analysis and PPI network.

26 samples in the GSE66271 (M1) dataset and 28 in the GSE66270 (M0) dataset were downloaded, with tumors and paracancerous tissues accounting for half of each dataset. All gene expression data were normalized (Figure 1A, 2A) and filtered with |log2FC > 2| and adj. *p*-value <0.05. Volcano plots (Figure 1B, 2B) were used to assess DEG gene expression broadly, and PCA analysis (Figure 1C, 2C) with a heatmap (Figure 1D, 2D) was used to assess differences in the expression patterns of tumor and paracancerous tissue patterns. Under these conditions, 355 upregulated and 554 downregulated genes were collected from the M1 dataset (Supplementary Table S2), and 630 upregulated and 805 downregulated genes were collected from the M0 dataset (Supplementary Table S3). Genes differentially expressed before and after tumor metastasis were obtained by taking the intersection of up and downregulated genes in the M0 and M1 datasets, respectively. The up and downregulated DEGs were then intersected with metabolic genes from the KEGG database (Supplementary Table S1) to obtain DEMGs expressed in the tumor before and after metastasis. 17 and 72 up and downregulated DEMGs were obtained (Supplementary Table S4), respectively. This process was visualized with a Venn diagram (Figure 3).

**FIGURE 4:**
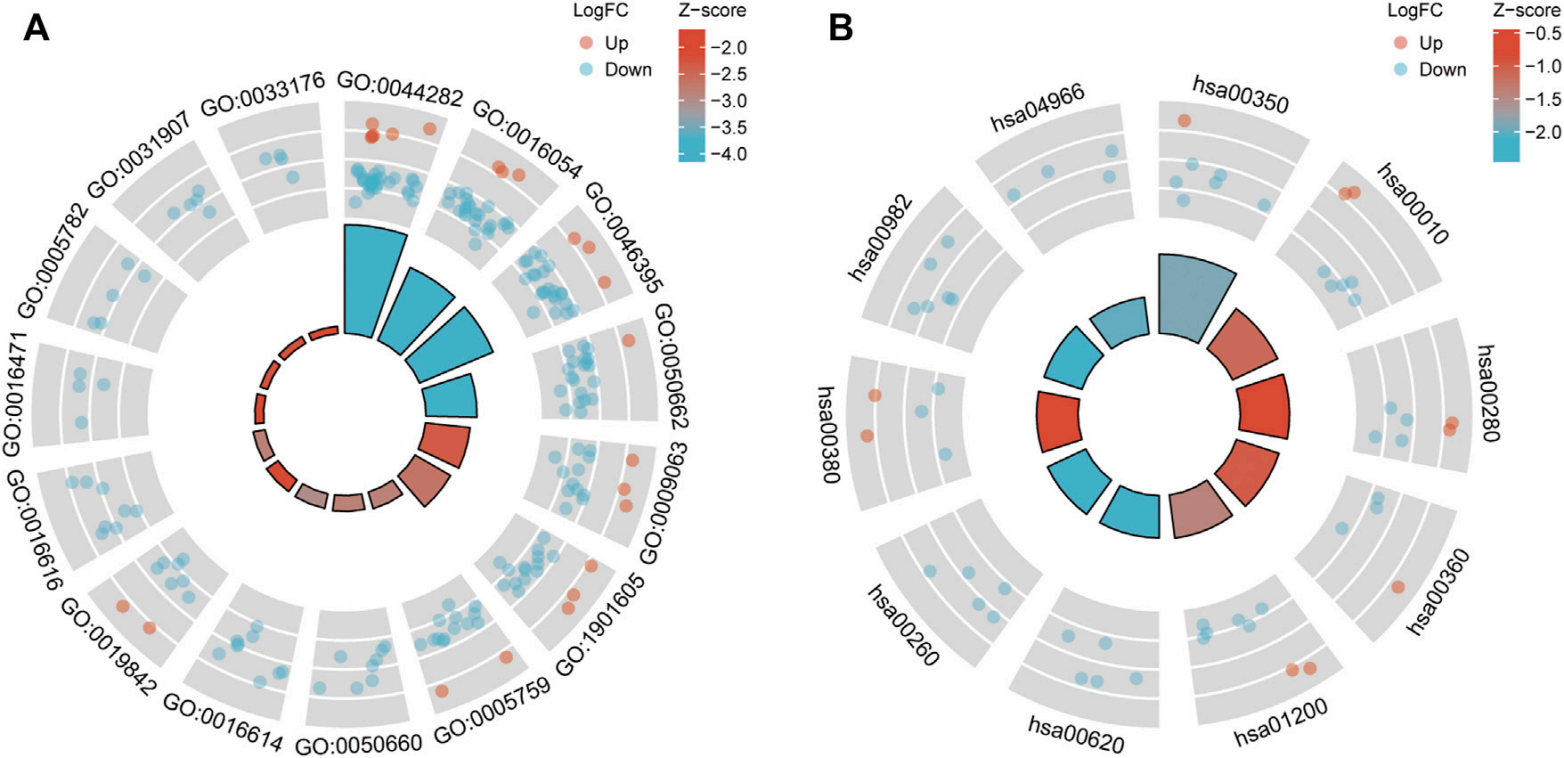
Results of GO enrichment **(A)** and KEGG pathway analyses **(B)**.

GO (Figure 4A) and KEGG pathway enrichment (Figure 4B) analyses were performed to clarify the DEGs’ potential biological processes and signaling pathways. DEG expression was determined using the M1 dataset, and the data (Supplementary Tables S5, S6) were visualized. The plot can be divided into the inner and outer circles. Each column of the inner circle corresponds to an entry, and the height is the relative size of the adj. *p*-value. The higher the value, the smaller the p. adjust of the ID. The color of the corresponding column represents the Z-score value of the entry. “Up” and “Down” represent the logFC of the molecules corresponding to the entry as positive and negative.

**FIGURE 5:**
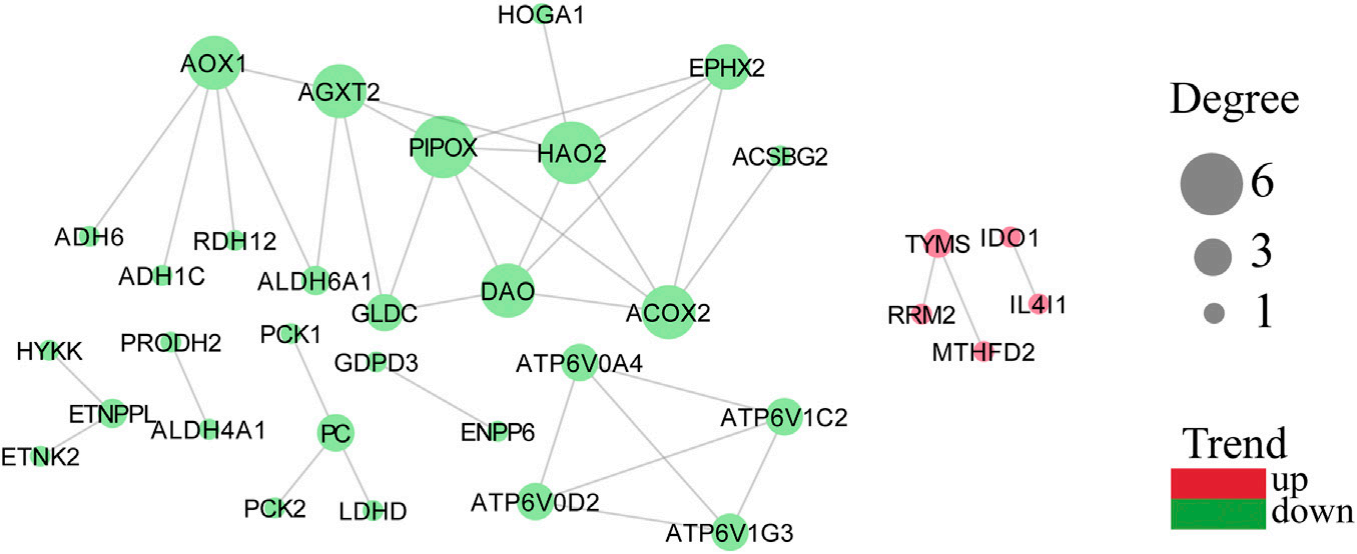
PPI network with node degree value with trends. A more considerable degree value of a target represents more association with other targets. Green represents downregulation and red represents upregulation.

Protein coding genes undergo transcription and translation to produce corresponding proteins with genetically determined functions. In contrast, the execution of protein function is not isolated, and there is a mutual connection between individual proteins. The STRING database collected PPI information from the DEMGs, and the “highest confidence (0.900)” was set as the required minimum interaction score. The data were imported into Cytoscape (v3.8.0) for network visualization. The color of the network node represents whether a gene is up or downregulated, and the size of the network node correlates positively with the degree of the node (Figure 5).

### 3.2 Result of Cox Regression Analysis and Screened Prognostic Related DEMGs

**FIGURE 6:**
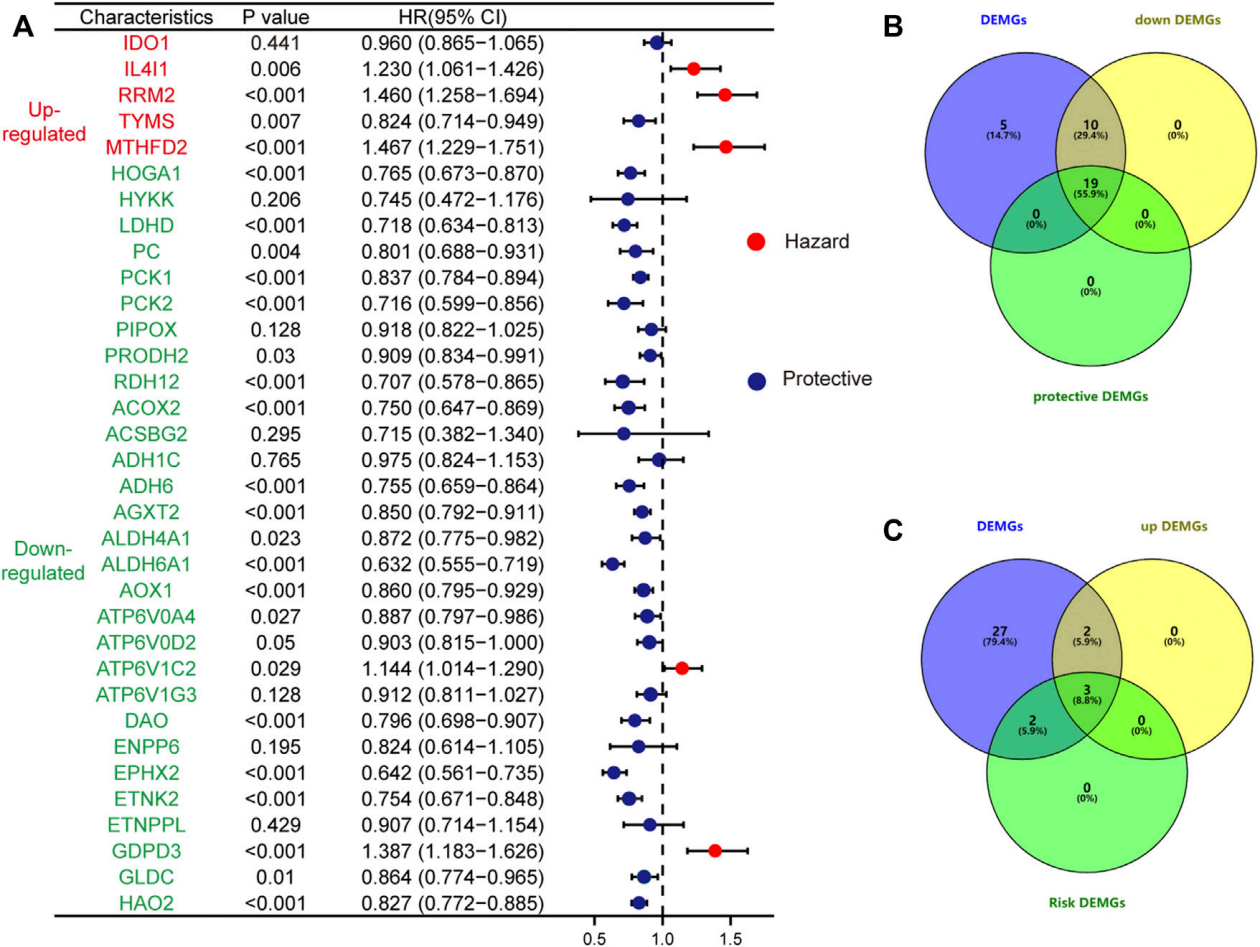
The forest plot shows the results of the univariate Cox regression analysis **(A)**; The up-regulated risk genes **(B)** and down-regulated protective genes **(C)** in DEMGs were screened by taking the intersection.

At a screening conditional *p*-value <0.05, univariate Cox regression analysis results showed that 5 up and 29 downregulated DEMGs were associated with patient prognosis, and gene-disease associations became smaller when the HR approached 1. High expression of risk genes (HR > 1) was associated with poor prognosis, whereas high expression of protective genes (HR < 1) was associated with good prognosis (Figure 6A). The risk genes that were upregulated and protective genes that were downregulated in tumors were of particular interest. By intersection screening, 3 upregulated risk genes and 19 downregulated protective genes were obtained from the DEMGs (Figure 6B, C).

**FIGURE 7:**
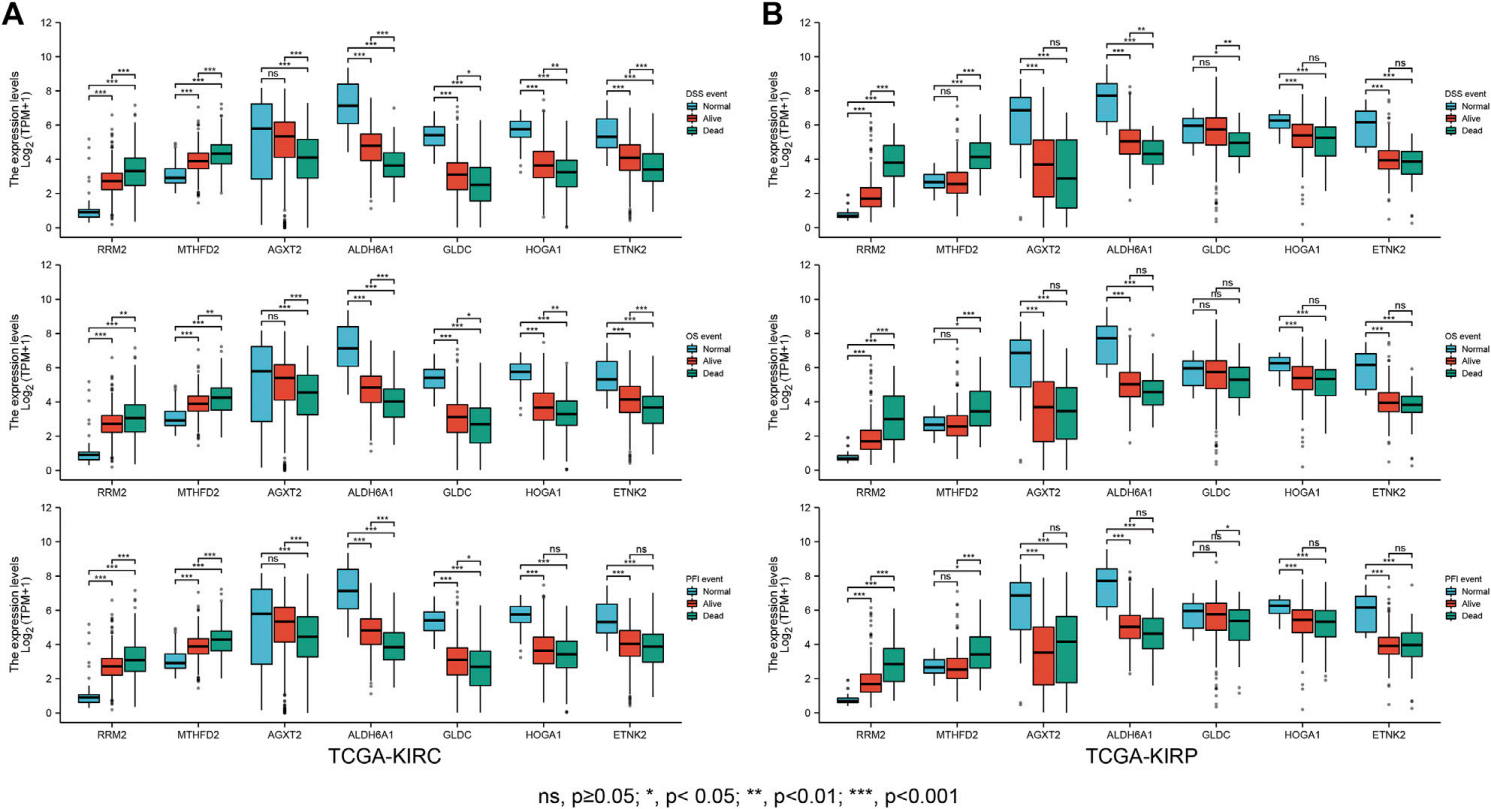
Clinical outcome correlation analysis of the DEMGs. Boxplots showed that DEMG expression differed significantly between patients with different clinical outcomes. Compared with normal individuals, patients in the TCGA-KIRC cohort **(A)** and TCGA-KIRP **(B)** whose clinical outcome was survival had upregulated risk gene expression and downregulated protective gene expression. This trend was more pronounced in patients with a clinical outcome of death.

To further screen and explore the relationship between the obtained genes and prognosis, the expression of each gene in TCGA-KIRC patients was assessed, duplicates were excluded, and the gene expression levels were divided into a high expression (high) and low expression (low) group according to the median expression level. The patients’ differential OS, DSS, and PFI were analyzed by Cox regression to plot the KM survival curves; Screening resulted in 16 prognostically relevant DEMGs (Table 1) (Supplementary Figures S1, S2). The expression of potential biomarkers under different survival outcomes was explored in the TCGA-KIRC cohort. High expression of risk genes and low expression of protective genes correspond with worse prognostic outcomes (Figure 7A). pRCC is the second most prevalent phenotype among RCC, and similar results were found in the TCGA-KIRP cohort (Figure 7B).

**TABLE 1:** Significance testing for survival analysis.

Group	Tendency	DSS *p*-value	OS *p*-value	PFI *p*-value
IL4I1	Up	*p* = 0.143	*p* = 0.264	*p* = 0.107
MTHFD2	Up	*p* = 0.001	*p* = 0.002	*p* < 0.001
RRM2	Up	*p* = 0.002	*p* = 0.002	*p* = 0.002
ACOX2	Down	*p* < 0.001	*p* = 0.005	*p* < 0.001
AGXT2	Down	*p* < 0.001	*p* < 0.001	*p* < 0.001
ALDH6A1	Down	*p* < 0.001	*p* < 0.001	*p* < 0.001
ALDH4A1	Down	*p* = 0.048	*p* = 0.05	*p* = 0.245
ADH6	Down	*p* = 0.001	*p* = 0.002	*p* = 0.001
AOX1	Down	*p* < 0.001	*p* < 0.001	*p* = 0.058
GLDC	Down	*p* = 0.001	*p* = 0.001	*p* = 0.001
HOGA1	Down	*p* = 0.009	*p* = 0.002	*p* = 0.024
RDH12	Down	*p* < 0.001	*p* = 0.001	*p* = 0.006
ATP6V0A4	Down	*p* = 0.505	*p* = 0.694	*p* = 0.22
DAO	Down	*p* < 0.001	*p* < 0.001	*p* = 0.009
EPHX2	Down	*p* < 0.001	*p* < 0.001	*p* < 0.001
ETNK2	Down	*p* < 0.001	*p* < 0.001	*p* = 0.015
HAO2	Down	*p* < 0.001	*p* < 0.001	*p* < 0.001
LDHD	Down	*p* < 0.001	*p* < 0.001	*p* < 0.001
PC	Down	*p* = 0.098	*p* = 0.289	*p* = 0.123
PCK1	Down	*p* < 0.001	*p* < 0.001	*p* < 0.001
PCK2	Down	*p* < 0.001	*p* < 0.001	*p* = 0.003
PRODH2	Down	*p* = 0.054	*p* = 0.054	*p* = 0.272

**FIGURE 8:**
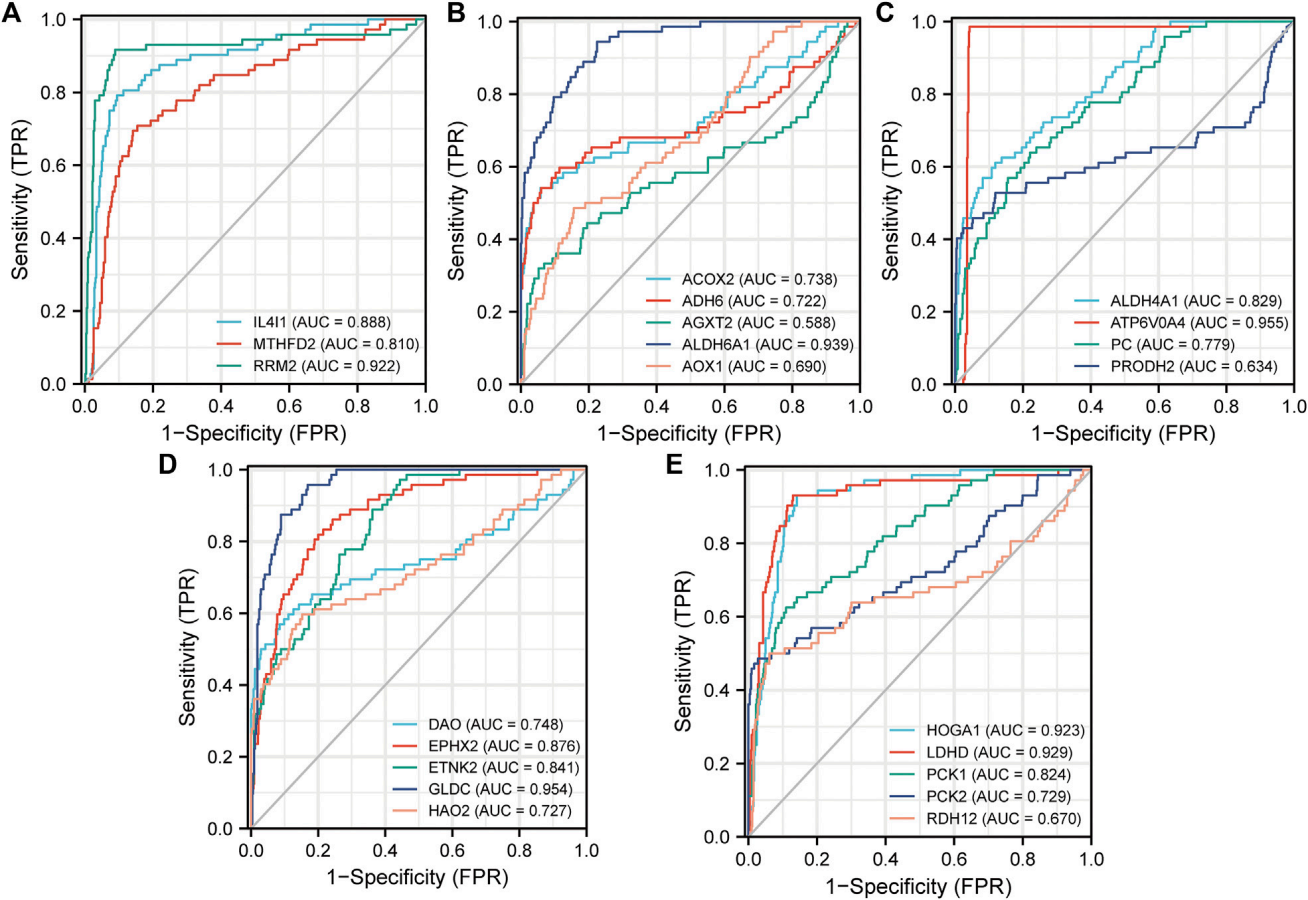
ROC curves for the 3 upregulated risk genes **(A)** and the 19 downregulated protective genes **(B)**–**(E)**. Construction and Evaluation of the MRSS Prognostic Value.

Delong’s test was used to test the prediction accuracy of independent DEMGs, and the results were visualized with the pROC package. The area under the ROC curve range (AUC) ranged from 0.5 to 1. The closer the AUC is to 1, the higher the detection accuracy. Conversely, an AUC equal to 0.5 suggests low accuracy of detection. The selected DEMGs all had high accuracy and could predict ccRCC outcomes (Figure 8).

**FIGURE 9:**
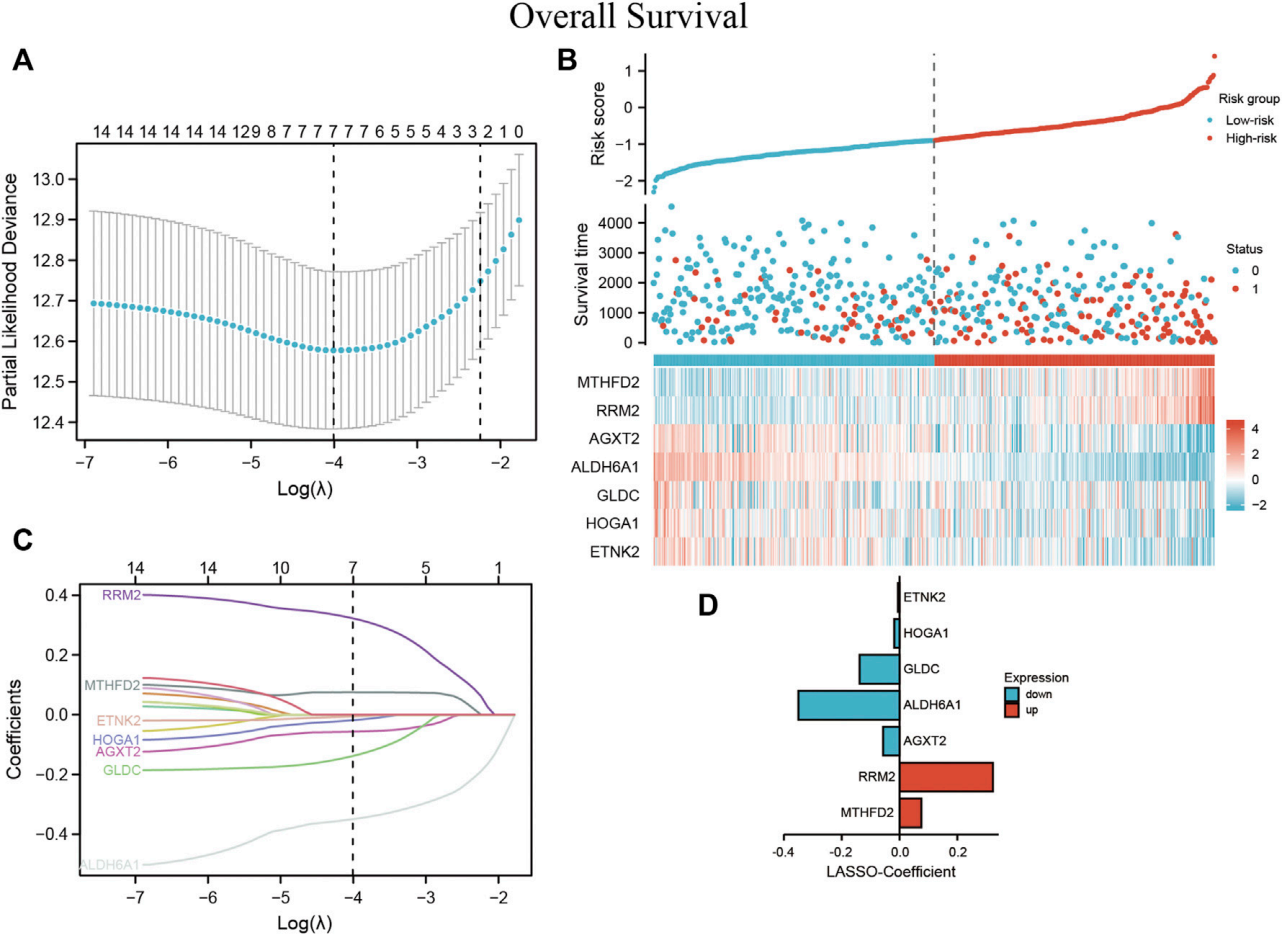
MRSS built using ccRCC patient data **(A)** Optimization of the model parameters by 10-fold cross-validation. **(B)** The risk score, survival status, and heatmap of 7 DEMGs in the TCGA-KIRC patient cohorts **(C)** Lasso coefficient profiles of the 16 DEMGs from the survival analysis and **(D)** coefficient value barplot of each model gene.

Lasso Cox regression analysis was performed to test whether the screened DEMGs could serve as a prognostic biomarker for ccRCC (Figures 9A,B). The model achieves the best prediction when 7 is chosen as the penalty coefficient (Figure 9C) (Supplementary Tables S7, S8). We also performed multivariate Cox regression analysis on the 7 metabolism genes, which were still able to enter the equation as a prognostic predictor (Supplementary Table S9). The corresponding regression coefficients of 7 metabolic genes, MTHFD2, RRM2, AGXT2, ALDH6A1, GLDC, HOGA1, and ETNK2 were 0.075, 0.323, - 0.057, - 0.350, - 0.138, - 0.018 and -0.006, respectively (Figure 9D). The formula of the established MRSS was as follows:

MRSS=ExpMTHFD2∗0.075+ExpRRM2∗0.323+ExpAGXT2∗−0.057+ExpALDH6A1∗−0.350+ExpGLDC∗−0.138+ExpHOGA1∗−0.018+ExpETNK2∗−0.006

### 3.3 The Value of MRSS in Predicting Clinical Characteristics

**FIGURE 10:**
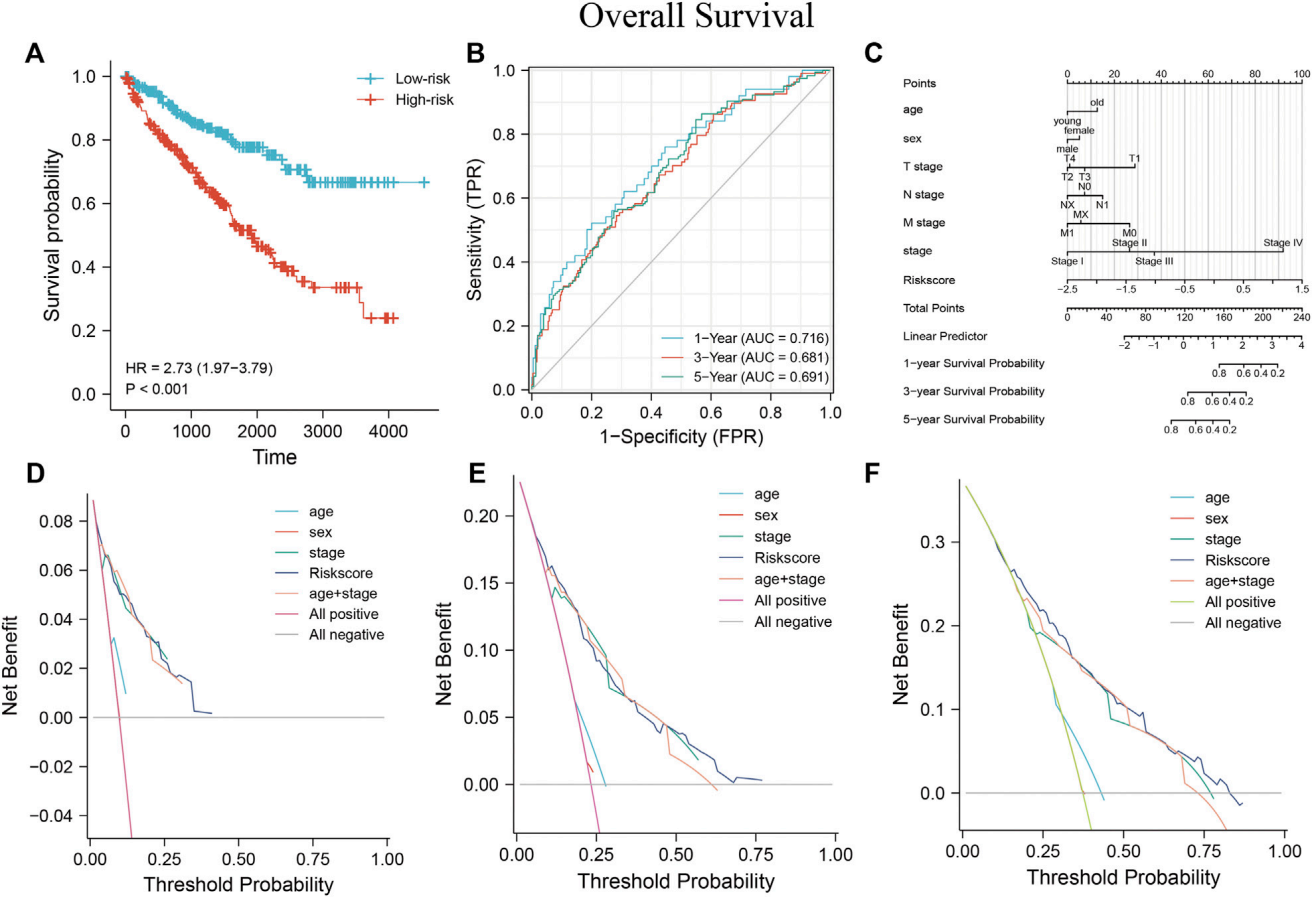
Establishment of MRSS and assessment of its predictive value using nomograms **(A)** OS survival curves were significantly different between the high- and low-risk groups in the TCGA-KIRC dataset. **(B)** Time-dependent ROC curves showed that MRSS predicted patients 1 -, 3 - and 5-years OS with sufficient accuracy **(C)** Nomogram for predicting patient outcome in the TCGA-KIRC cohort incorporating multiple clinicopathologic factors. DCA curves to examine the clinical application of MRSS, nomogram, and independent clinicopathological factors at 1- **(D)**, 3- **(E)**, and 5-years **(F)**.

Based on the MRSS formula, the risk score of each patient in the TCGA-KIRC cohort was collected, and the patients were subsequently divided into high-risk and low-risk groups based on the median score. Results from the KM survival curve for the prognosis of patients in the high-risk group performed worse than those in the low-risk group (Figure 10A). ROC curves were plotted to evaluate the ability of the established models to predict patient outcomes at 1, 3, and 5 years and the AUC values were 0.716, 0.681, and 0.691, respectively, indicating that the established models were able to predict patient outcomes (Figure 10B).

Univariate and multivariate Cox regression analyses determined whether the established MRSS had prognostic significance. The univariate Cox regression analysis showed that risk scores, TNM stage, cancer stage, and serum calcium concentration may be reliable prognostic indicators. Furthermore, the risk score was the only significant predictor in the multivariate Cox regression analysis. These results suggested that the established MRSS model could be a valuable biomarker for predicting ccRCC outcomes (Table 2).

**TABLE 2:** Univariate and multivariate Cox regression analysis of clinicopathological factors associated with OS of ccRCC patients.

Variables		Patient N (539)	Univariate Analysis		Multivariate Analysis	
			HRa [95% CI]	P	HR [95% CIb]	P
Age	Young	250	1			
	old	289	1.802 [1.315,2.468]	<0.001*	0.992 [0.679,1.452]	0.969
Sex	male	353	1			
	female	186	1.075 [0.788,1.465]	0.648		
T stage	T1	278	1			
	T2	179	1.515 [0.908,2.526]	0.112	0.341 [0.104,1.120]	0.076
	T3	71	3.354 [2.373,4.742]	<0.001*	0.488 [0.181,1.316]	0.156
	T4	11	10.829 [5.467,21.451]	<0.001*	0.519 [0.166,1.620]	0.259
N stage	N0	241	1			
	N1	16	3.565 [1.895,6.705]	<0.001*	1.320 [0.579,3.010]	0.510
	NX	282	0.818 [0.601,1.114]	0.203	0.773 [0.562,1.063]	0.113
M stage	M0	428	1			
	MX	31	0.828 [0.262,2.615]	0.748	0.495 [0.124,1.967]	0.193
	M1	78	4.400 [3.219,6.014]	<0.001*	0.417 [0.112,1.555]	0.318
Stage	Stage I	272	1			
	Stage II	59	1.210 [0.652,2.247]	0.546	2.739 [0.715,10.494]	0.142
	Stage III	123	2.711 [1.804,4.073]	<0.001*	1.888 [0.593,6.013]	0.282
	Stage IV	83	6.782 [4.633,9.929]	<0.001*	4.894 [0.872,27.448]	0.071
Riskscore		537	2.718 [2.297,3.217]	<0.001*	2.663 [1.739,4.079]	<0.001*

aHR, hazard ratio; bCI, confidence interval. *p < 0.05.

The nomogram is a standard clinical tool to evaluate patient prognosis, combining different prognostic factors and variables to comprehensively assess the probability of clinical events within a certain period. Compared with traditional disease staging, the user-friendly nomogram brings higher accuracy and is more accessible to understand prognoses by digitizing various factors and simple calculations (Balachandran et al., 2015). Several factors showed prognostic correlation, so a nomogram containing a variety of pathological factors was established, including the MRSS model. The nomogram (Figure 10C) showed that many prognostic factors were digitally assigned, and ccRCC patient outcomes could be reliably predicted by calculating the score.

The consistency index (c-index) refers to the proportion of all patient pairs whose predicted results are consistent with actual observations. The c-index was used to evaluate the predictive ability of various characteristics. The nomogram c-index that combined multiple clinicopathological factors was the highest at 0.771 and slightly weaker at MRSS, with a c-index of 0.769 compared with 0.755 in the conventional TNM stage c-index (Table 3). As a result, the model outperformed conventional TNM stage prediction but was weaker than the comprehensive nomogram. Consistent with the c-index, the nomogram that incorporated multiple clinicopathological factors performed best in the DCA curve (Figures 10D–F).

**TABLE 3:** C-index.

Cancer Species	Variables	C-Index (95%CI)
KIRC	TNM-stage	0.755 (0.729–0.781)
	MRSS	0.769 (0.750–0.787)
	nomogram	0.771 (0.753–0.790)
KIRP	TNM-stage	0.573 (0.530–0.616)
	MRSS	0.758 (0.716–0.801)
	nomogram	0.842 (0.802–0.881)

### 3.4 Validation of Other Kidney Cancer Species

According to the WHO classification of urinary cancers, ccRCC, characterized by malignant tumors composed of clear or eosinophilic cytoplasmic cells, is the largest pathological renal cancer subtype, accounting for 60–85% of cases. The second most common RCC is pRCC, which originates from tubular epithelial cells and accounts for 18.5% of reported RCC cases.

The model in this study was validated using representative pRCC and clinical data from the TCGA-KIRP cohort. A risk score was calculated for each patient in the TCGA-KIRP cohort using the previously established MRSS formula. The cohort was divided into high- and low-risk groups based on the median patient risk score.

**FIGURE 11:**
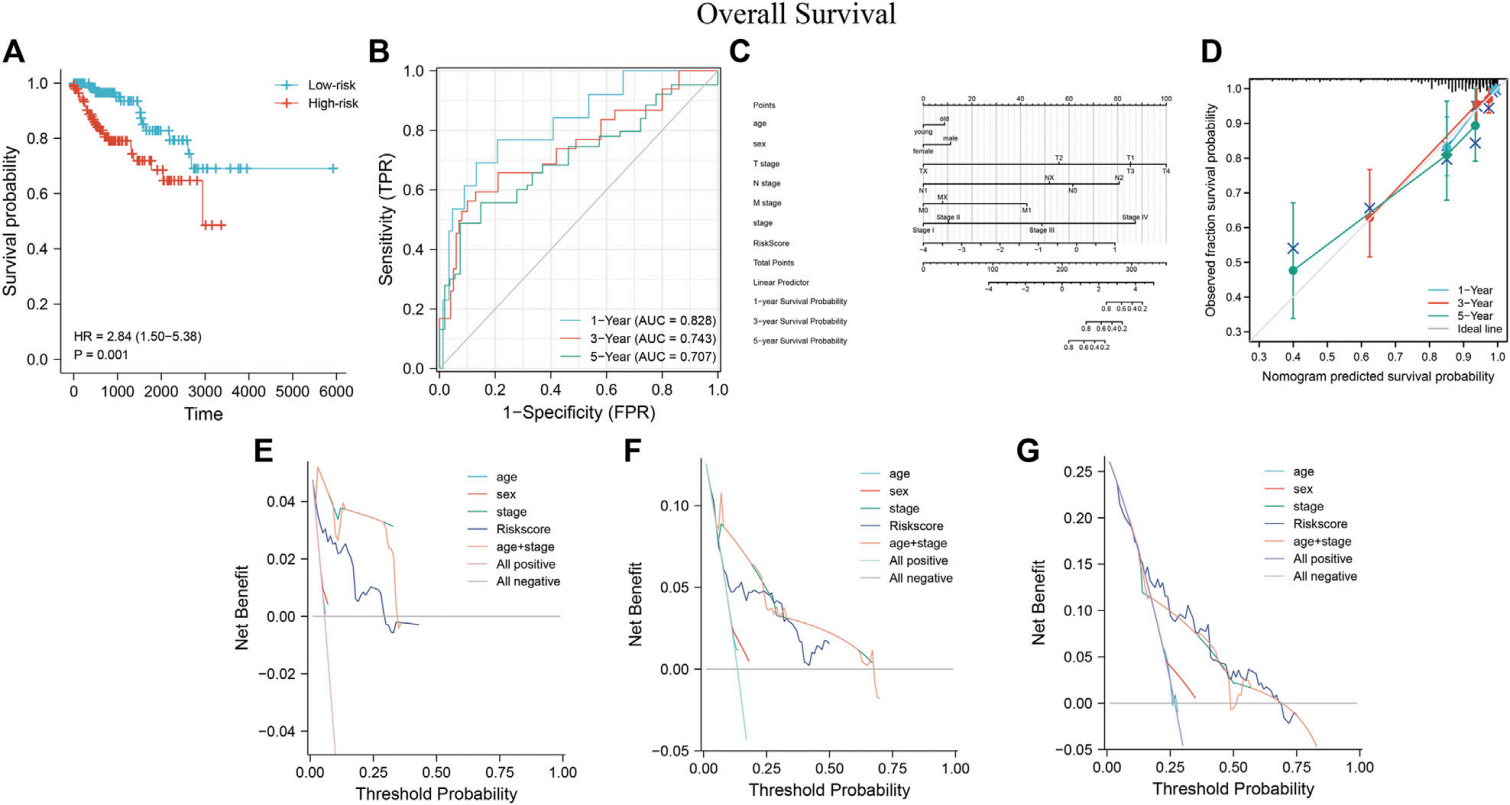
The predictive performance of MRSS using the TCGA-KIRP cohort **(A)** OS survival curves show that the low-risk group has better prognostic outcomes than the high-risk group. **(B)** Time-dependent ROC curve analysis of MRSS at 1, 3, and 5 years **(C)** Nomogram used to predict 1-, 3-, and 5-years survival probabilities for patients in the TCGA-KIRP cohort. **(D)** Calibration curves were used to evaluate the fitting effect of the nomogram on the prediction of patient survival probability at 1, 3, and 5 years with the actual outcomes. DCA curve of the nomogram, MRSS, and pathology-based tumor staging to evaluate the survival prediction of patients in the TCGA-KIRP cohort at 1**(E)**, 3**(F)**, and 5**(G)** years.

Survival analysis showed that the low-risk group had a better prognosis than the high-risk group (Figure 11A) (*p* = 0.001; HR = 2.84, 95% CI = 1.50–5.38). Time-dependent ROC analysis curves showed good agreement between the actual OS of the model built-in patients in predicting OS (Figure 11B, 0.828, 0.743, and 0.707 in 1, 3, and 5 years, respectively). A predictive nomogram was developed using standard clinical features and MRSS for predicting the likelihood of 1, 3, and 5-years prognostic survival in patients with pRCC (Figure 11C). The calibration curve showed that the predictive effect of the model on patient survival outcomes fitted well with the actual observation (Figure 11D). C-index and DCA curve results consistently revealed that the predictive effect of MRSS on the prognostic survival probability of pRCC was better than that of conventional TNM staging, and the nomogram had the best predictive effect (Figures 11E–G).

**FIGURE 12:**
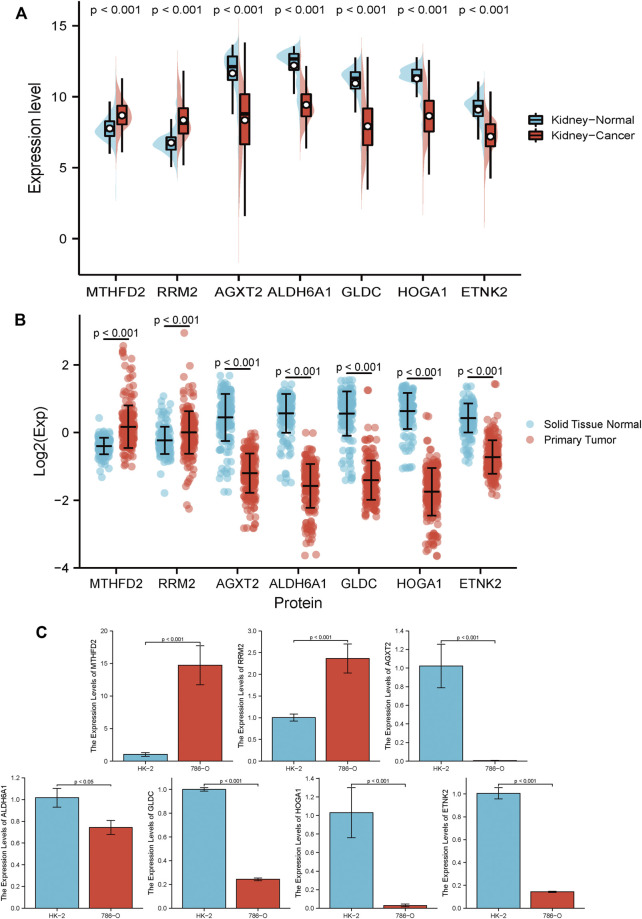
RCC biomarker expression at the transcript and protein level **(A)** 7 independent biomarkers had different transcript expressions in tumor and normal tissues. **(B)** 7 independent biomarkers had different protein expression in tumor and normal tissues. **(C)** 2 risky genes, RRM2 and MTHFD2, significantly increased in 786-O cells compared with HK-2 cells at the transcript level. The remaining 5 protective genes, AGXT2, ALDH6A1, GLDC, HOGA1, and ETNK2, were significantly down-regulated in 786-O cells compared to HK-2 cells at the transcript level. All results are expressed as mean ± SD, *n* = 6 per group.

To further investigate the prognostic value of the established model, GENT2 (Figure 12A) and CPTAC (Figure 12B) databases were used to examine the expression of each gene in the MRSS model at the transcription and protein levels; we also tested transcript levels in cultured cells (Figure 12C). Consistent with the TCGA database, the respective genes composing MRSS maintained similar transcription and protein expression. The GEPIA database (Supplementary Figure S3A) and Kaplan-Meier plot (Supplementary Figure S3B) results showed that the expression of each gene in MRSS was highly correlated with the prognosis of ccRCC patients, and the respective independent genes could serve as potential biomarkers.

## 4 Discussion

RCC, also known as renal adenocarcinoma, is a highly malignant tumor in the urinary system that accounts for 80–90% of malignant renal tumors. This disease has a high degree of heterogeneity, because it is associated with mild symptoms in its early stages and is thus usually diagnosed and treated once it has advanced (Hsieh et al., 2017; Motzer et al., 2022). Kidney cancer is the third most common cancer of the genitourinary system, second only to prostate and bladder cancer, accounting for 2–3% of malignant tumors in adults (MacLennan et al., 2012). The incidence and mortality of RCC are rising each year, drawing worldwide attention (Mokdad et al., 2017).

Past studies have explored the molecular mechanisms of common RCC subtypes such as ccRCC and pRCC. The findings suggest that genes associated with kidney cancer, including VHL, MET, FH, FLCN, TSC1, TSC2, and SDH, are involved in metabolic pathways linked to oxygen and iron or nutrient sensing, thus characterizing kidney cancer as a cellular metabolic disease (Linehan et al., 2010). Indeed, the carcinogenic process of ccRCC, the primary subtype of RCC, is closely related to metabolism. A hallmark of ccRCC is metabolic remodeling, including stabilization of HIF1/2 transcription factors resulting from VHL mutations, which create pseudohypoxia, increase glycolysis and angiogenic growth factor secretion, and elevate expression of proteins, including CCND1, PDK1, LDH, and GLUT1, that are associated with glucose metabolism regulation and cell proliferation (Linehan et al., 2010; Linehan et al., 2019; Xiao et al., 2020).

More patients show no significant prognostic improvement following applied tumor immunotherapy, suggesting that reliable biomarkers for predicting treatment outcomes should remain a significant focus of oncology research efforts (Motzer et al., 2018; Motzer et al., 2019; Rini et al., 2019; Braun et al., 2021). The current study aims to establish reliable biomarkers associated with metabolism to effectively predict the prognostic outcome of ccRCC patients. The role of metabolism-related genes in the development and metastasis of ccRCC was used to construct a prediction model by analyzing and validating it using the TCGA-KIRP cohort and external datasets. Since the two pathological subtypes, ccRCC, and pRCC, account for more than 90% of RCC case reports, they were used to assess the efficacy of the established prediction model in RCC.

Human-derived genes using metabolic terms were downloaded from the KEGG database, and the GSE66272 dataset was downloaded from GEO. Since the GEO dataset has pre- and post-tumor metastasis subsets, we separated these into the GSE66270 and GSE66271 datasets to find DEGs. These genes were intersected to obtain differentially expressed before and after metastasis. The DEGs were crossed with metabolism-related genes to obtain DEMGs before and after tumor metastasis. GO and KEGG pathway enrichment analysis results showed that DEMGs were associated with multiple metabolism-related biological processes and pathways. The TCGA-KIRC cohort was then selected as the data cohort to elucidate the correlation between metabolism and ccRCC. Using univariate Cox regression analysis on the DEMGs, the HRs of DEMGs for ccRCC were obtained. Of these, upregulated risk genes and downregulated protective genes were selected to coincide with tumor progression.

The prognosis-related ROC curves of each gene were plotted to assess the relationship between prognosis-relevant DEMGs and the OS, DSS, and PFI by KM patient survival curves. The prognosis-related DEMGs were selected for further model construction. MRSS predictive prognosis was developed using Lasso-Cox regression analysis of the selected genes. These included the RRM2 gene, which encodes a ribonucleotide reductase subunit and provides raw materials for DNA synthesis. Deregulated cell proliferation dramatically increases DNA replication in cancer cells, increasing the demand for raw materials needed for DNA synthesis. Studies indicate that RRM2 is involved in cytogenetic material synthesis and promotes the growth and metastasis of various cancers (Dawany et al., 2011; Das et al., 2021). In contrast, silencing RRM2 gene expression induced cell cycle arrest and inhibited cell proliferation (Yang et al., 2017). Moreover, RRM2 promoted RCC cell acquired resistance to VEGF tyrosine kinase inhibitors, inhibiting the effect of PD-1 blocker immunotherapy (Xiong et al., 2021).

MTHFD2 encodes a bifunctional methylenetetrahydrofolate dehydrogenase/cyclohydrolase, a mitochondrial enzyme that participates in one-carbon metabolism, and studies indicate that in RCC, MTHFD2 can remodel metabolism through RNA methylation. In contrast, knockdown of MTHFD2 expression reduced xenograft tumor growth (Green et al., 2019). This may be associated with modulation of the NADPH to NADP ratio in cancer cells, depleting GSH, and triggering cancer cell apoptosis (Yang et al., 2022). MTHFD2 is also a metabolic checkpoint linking purine metabolism to autoimmune responses (Sugiura et al., 2022). It is highly expressed in various cancers, playing a role in metabolic remodeling and regulating of the cell cycle in the mitochondria and nucleus, respectively (Lee et al., 2021b; Liu et al., 2021; Yao et al., 2021; Ren et al., 2022).

AGXT2 is a multifunctional mitochondrial aminotransferase with diverse cellular physiological functions predominantly expressed in kidney cells and hepatocytes. Its substrates are biomarkers for renal, cardiovascular, and metabolic diseases (Rodionov et al., 2014; Ye et al., 2021).

ETNK2 is an ethanolamine kinase that has enhanced expression in gastric, non-small cell lung, and prostate cancers (Miwa et al., 2021). ETNK2 promotes liver metastasis of gastric cancer by inhibiting the p53-Bcl2 apoptotic pathway, resulting in a poor prognosis. Phosphatidylethanolamine synthesis in non-small cell lung cancer is significantly enhanced by ETNK2, whereas reduced ETNK2 expression in prostate cancer results from the loss of TET2 targeted demethylation (Kamdar et al., 2019; Lesko et al., 2021).

HOGA1 encodes the mitochondrial 4-hydroxy-2-oxoglutarate aldolase, and mutations cause oxalate accumulation in the kidney and primary hyperoxaluria type 3 (Ventzke et al., 2017).

GLDC primarily regulates glycine metabolism and is an essential metabolic enzyme for protein and amino acid metabolism. GLDC also promotes non-small cell lung cancer progression by inducing glycolysis with pyrimidine metabolism. GLDC inhibition impairs pyruvate metabolism in cancer cells, resulting in loss of their metabolic energy source (Zhang et al., 2012; Woo et al., 2018). GLDC upregulation induces autophagy in HCC cells and inhibits liver cancer metastasis. High GLDC expression in neuroblastoma cells prevents the accumulation of toxic metabolites. In contrast, GLDC inhibition in glioblastoma causes an accumulation of glycine and results in reduced cell viability, indicating that glycine catabolism by GLDC is critical for proliferation and tumorigenesis (Alptekin et al., 2019; Abdollahi et al., 2021). However, GLDC was significantly decreased in ccRCC, while overexpression suppressed the proliferation and migration of tumor cells (Chen et al., 2020).

Previous studies indicate that the ALDH6A1 gene is related to the aldehyde dehydrogenase family of proteins, and the mitochondrial methylmalonate semialdehyde dehydrogenase encoded by ALDH6A1 functions in the valine and pyrimidine catabolic pathways. There is an inverse correlation between ALDH6A1 expression and both RCC progression and patient outcomes (Perroud et al., 2009). A similar trend was reported during collecting duct cancer and the progression of liver cancer (Wach et al., 2019; Shin et al., 2020). Studies have also shown that ALDH6A1 was positively expressed in breast cancer stem cells, and gradually decreased during tumor progression (Johansson et al., 2015; Xu et al., 2021). These results indicate that the same trends occur in the progression of different cancer types, suggesting that ALDH6A1 may be involved in tumor initiation and progression.

PRCC is the second most common RCC subtype, histologically resembling ccRCC in anatomic location and sharing many similar oncogenic factors (Guimarães-Teixeira et al., 2021; Tian et al., 2021; Weng et al., 2021). As a result of possible similarities in anatomical location, causative factors, and histological type, TCGA-KIRP data was used as a validation cohort to evaluate the predictive value of MRSS.

Results of the survival analysis were used to divide patients into a high-risk and low-risk group based on the median risk score calculated by MRSS. There was a significant difference in the OS of the two groups of patients, indicating that MRSS may have the potential to become an effective biomarker for predicting ccRCC and pRCC outcomes. Furthermore, the ROC curve drawn by Delong’s test results indicated that the model was in good agreement with the observed patient prognosis. The nomogram with age, gender, pathological stage, and TNM stage as covariates showed that the established model remained a reasonable independent predictor. To better decipher the power of the model in predicting disease outcomes, nomograms that combined multiple clinical variables were developed to score the survival probability of each patient. DCA curve and c-index results showed that MRSS had a higher predictive accuracy than traditional TNM staging for this outcome. However, nomograms that integrated multiple clinical variables still performed best. Interestingly, the MRSS constructed as a biomarker could reliably predict ccRCC and pRCC patient outcomes, indicating that the model was robust and broadly applicable. These findings may expand the horizons of RCC treatment.

In RCC and other carcinoma research, biomarkers for predicting outcomes have become widely used (Huang et al., 2016; Mo et al., 2018; Qu et al., 2018; Wei et al., 2019). At the time of preparation of this manuscript, the existing literature had established and published studies on metabolic risk models for ccRCC (Liu et al., 2020; Guo et al., 2021). The number of metabolic genes associated with prognosis was different from those shown here due to varying screening criteria. However, however, the RRM2 and ALDH6A1 genes were included in the results, indicating that the model was reliable and robust.

This study explored a potential association between metabolism and ccRCC. The predictive effect of the established biomarkers based on the seven metabolic genes was verified as reliable and stable for patients with ccRCC and pRCC. The established model could serve as an independent prognostic biomarker, provide potential therapeutic targets for the clinical treatment of RCC, including ccRCC and pRCC, and add a dimension for correlation studies.

## Data Availability

The datasets presented in this study can be found in online repositories. The names of the repository/repositories and accession number(s) can be found below: https://www.ncbi.nlm.nih.gov/geo/, GSE66270; https://www.ncbi.nlm.nih.gov/geo/, GSE66271; https://portal.gdc.cancer.gov/projects/TCGA-KIRC, TCGA-KIRC; https://portal.gdc.cancer.gov/projects/TCGA-KIRP, TCGA-KIRP; https://proteomic.datacommons.cancer.gov/pdc/study/PDC000127, PDC000127.
