# Supplementary figures and images for: A Cluster of Metabolic-Related Genes Serve as Potential Prognostic Biomarkers for Renal Cell Carcinoma
Source: Front Genet. 2022 Jul 7;13:902064. doi: 10.3389/fgene.2022.902064 (PMC9301649; doi:10.3389/fgene.2022.902064)

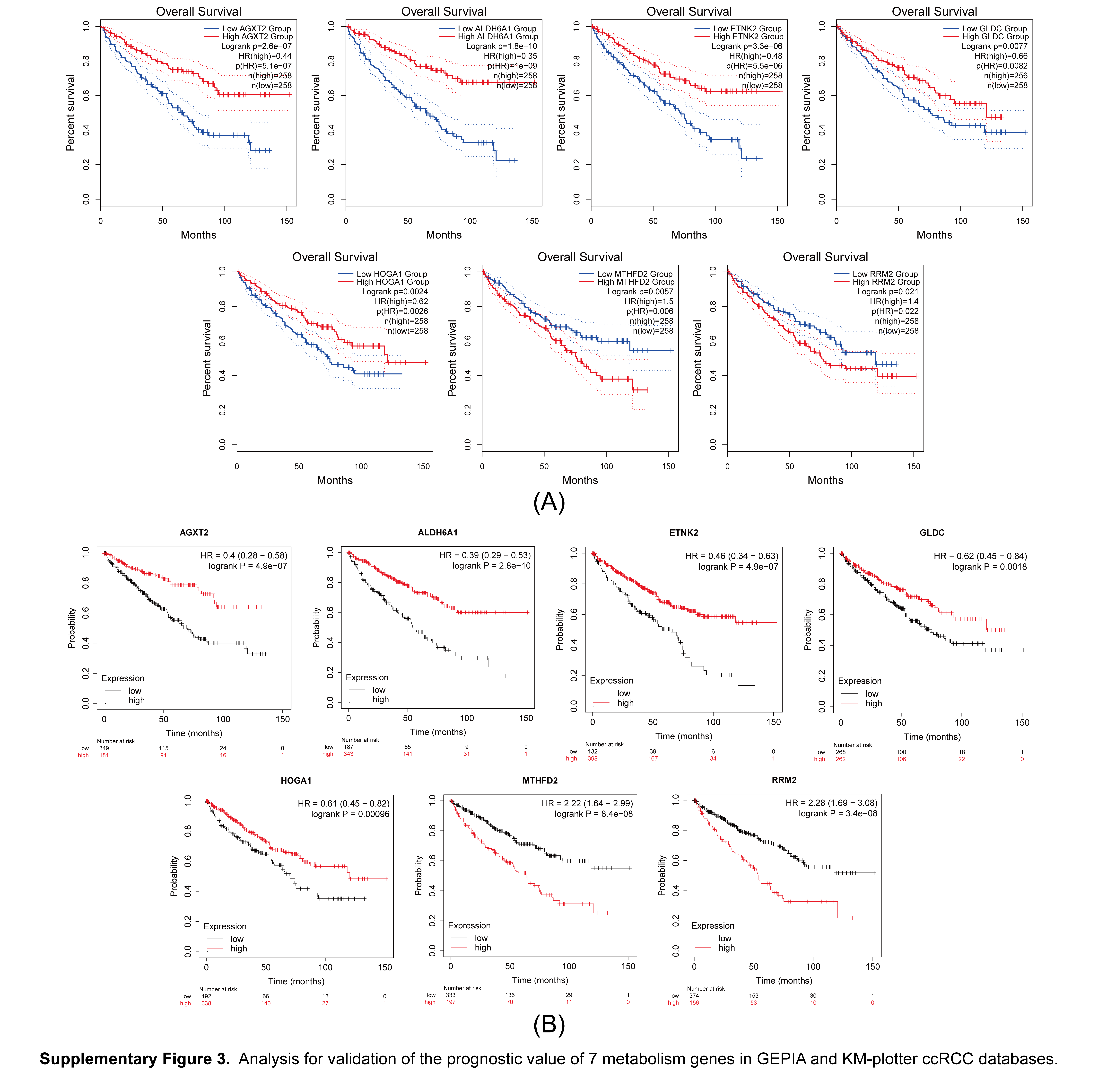

Supplement: Supplementary file 1 [file Image3.TIF]

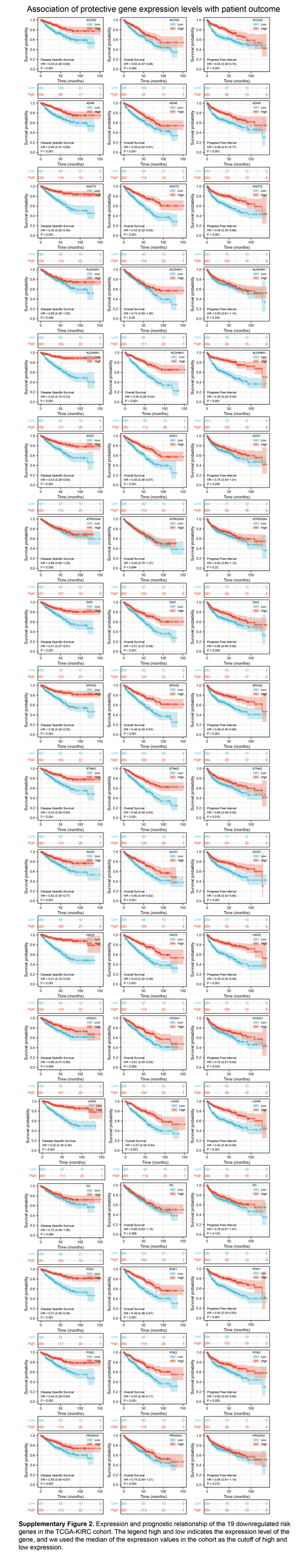

Supplement: Supplementary file 2 [file Image2.TIF]

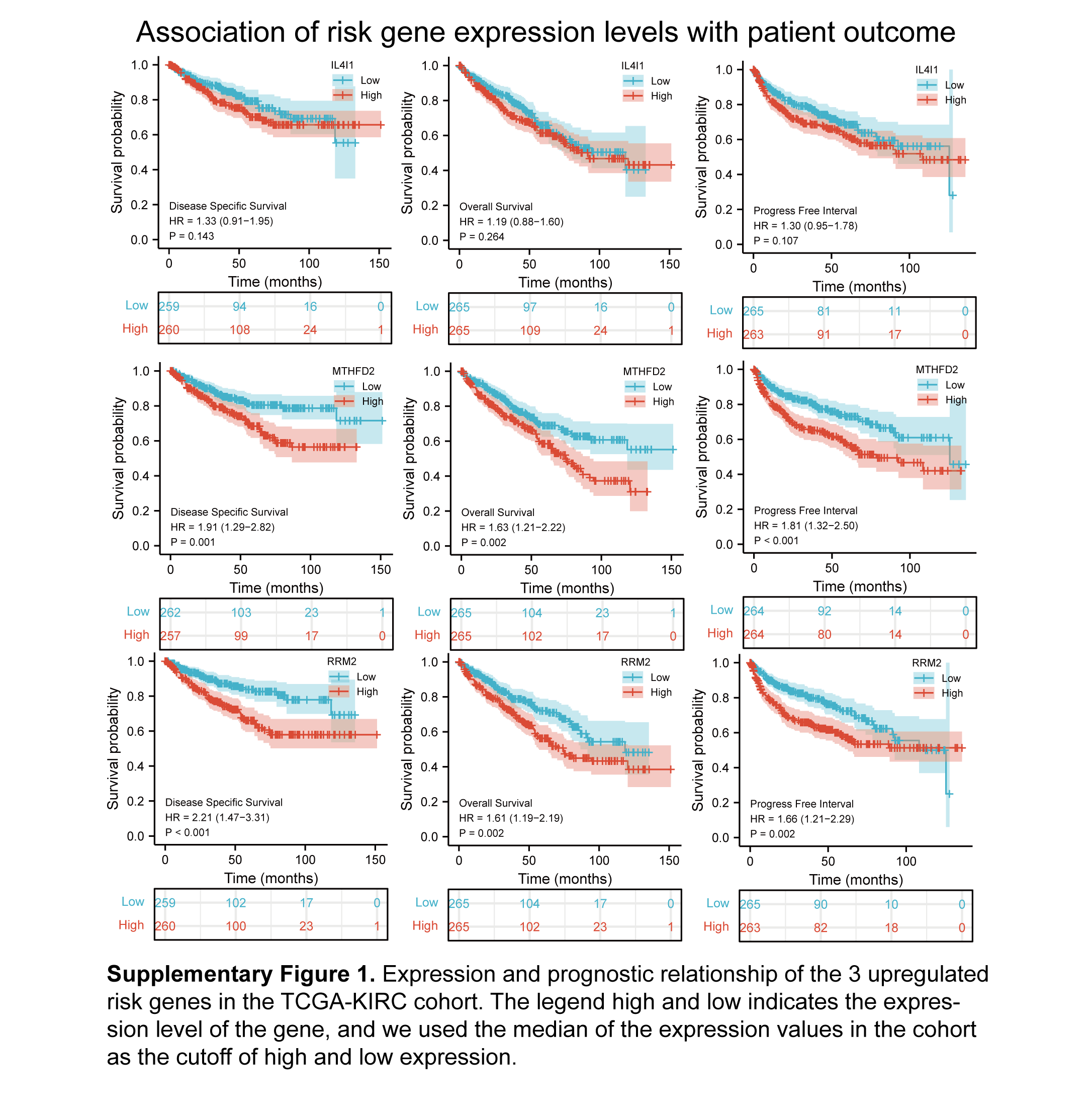

Supplement: Supplementary file 3 [file Image1.TIF]
